# Ethanol Extract From the Stem Bark of Adansonia digitata L. (Baobab) Displays Therapeutic Potential Against Klebsiella pneumoniae Infection

**DOI:** 10.7759/cureus.87520

**Published:** 2025-07-08

**Authors:** Germaine T Matsuete, Benjamin Tangue Talom, Stephen Lacmata Tamekou, Justine Odelonne Kenfack, Jean-De-Dieu Tamokou

**Affiliations:** 1 Department of Biochemistry, University of Dschang, Dschang, CMR; 2 Department of Biomedical Sciences, University of Ngaoundere, Ngaoundere, CMR

**Keywords:** adansonia digitata, klebsiella pneumoniae, mechanisms of action, pneumonia, therapeutic effect

## Abstract

Background

Pulmonary infection induced by *Klebsiella pneumoniae *is becoming one of the leading causes of respiratory infection worldwide. In recent years, the emergence of hypervirulent and multi-drug-resistant *K. pneumoniae *has been strongly associated with pneumonia*. *This study aimed to evaluate the *in vitro* and *in vivo* antibacterial properties of *Adansonia digitata *L. (Malvaceae), a medicinal plant traditionally used in the treatment of respiratory tract infections.

Methodology

Plant extract was obtained by maceration in ethanol. Qualitative chemical analysis of this extract was performed using standard methods. The *in vitro* antibacterial activities of the extracts alone and their combinations with Augmentin were evaluated using the broth microdilution method through the determination of Minimum Inhibitory Concentrations (MIC). The mechanisms of antibacterial action of the *A. digitata* extract were evaluated by targeting bacterial ATPases/H^+^ proton pump function, growth kinetics, outer membrane permeability, dehydrogenase activity, and leakage of nucleic acids. The therapeutic effect of the extract was evaluated *in vivo* on rats infected through the nasal cavity with *K. pneumoniae*.

Results

The phytochemical analysis of the *A. digitata* stem bark extract showed that it contains flavonoids, phenols, sterols, triterpenes, tannins, alkaloids, anthocyanins, anthraquinones, and saponins. The ethanol extract from *A. digitata* displayed *in vitro* antibacterial activities with MIC values ranging from 64 to 1024 μg/mL. *A. digitata* extract also displayed indifference (1 < fractional inhibitory concentration index (FICI) ≤ 4), additive (0.5 < FICI ≤ 1), and synergistic (FICI ≤ 0.5) effects with Augmentin that varied according to the tested bacteria. The plant extract induced changes in the bacterial growth kinetics, permeability of bacterial membranes, leakage of nucleic acids, and the inhibition of the bacterial ATPases/H^+^ proton pumps. Moreover, *A. digitata* extract showed to be active *in vivo* at the doses 5.00 and 9.00 mg/kg on *K. pneumoniae*-infected rats.

Conclusions

The findings demonstrate the *in vitro* antibacterial activity of *A. digitata* and its therapeutic efficiency against pneumonia induced by *K. pneumoniae*. Hence, *A. digitata* can be used for the development of phytomedicines to treat respiratory tract infections, particularly those caused by the tested bacteria. So, further studies are warranted.

## Introduction

*Klebsiella pneumoniae* is one of the most widespread pathogens worldwide. It remains the cause of high morbidity and mortality of respiratory tract infections and frequently affects elderly individuals as well as children under the age of five [1]. This bacterium can invade and cause serious infections in many sites, particularly in the lungs [2]. Its rate continues to worsen with many side effects due to common antibiotics and the multi-resistance of *K. pneumoniae* strains linked to the production of β-lactamase enzymes and/or broad-spectrum carbapenemases, leading to treatment failures with common antibiotics [3]. According to the World Health Organization (WHO), *K. pneumoniae* is classified as one of the priority and critical pathogens that require urgent research and development of new and effective antibiotics [4]. Antibiotic therapy has long been used to combat these microbial diseases. Higher plants produce hundreds of thousands of diverse chemical compounds with different biological activities and important ecological roles. This is the case of *Adansonia digitata* L. (Baobab), which contains many secondary metabolites that could justify its traditional use as antimicrobial, anti-inflammatory, antioxidant, antipyretic, analgesic, hepatoprotective, hypoglycemic, and hypolipidemic agents [5]. For instance, Vasconcelos et al. [6] have shown that compounds isolated from plant extracts have antibiotic potentiating activity against some bacterial strains, which cause lower respiratory tract infections (LRTIs). Likewise, Ács et al. [7] have shown that the essential oils of *Syzygium aromaticum*, *Cymbopogon nardus,* and *Mentha piperita* are sources of antibacterial agents against bacteria that cause respiratory tract infections. This is the case of *A. digitata,* which contains many secondary metabolites that could justify its traditional use in the treatment of respiratory infections [5]. In order to contribute to the search for plant extracts that can effectively fight against respiratory infections of bacterial origin, we have directed this study towards a medicinal plant traditionally used in the treatment of respiratory infections. Therefore, this study was aimed to evaluate the *in vitro* and *in vivo* antibacterial properties of *A. digitata*, a medicinal plant traditionally used in the treatment of respiratory infections, as well as its synergistic effect with Augmentin.

## Materials and methods

Plant material

The plant material used in this study consisted of the stem bark of *A. digitata* L. (Malvaceae) harvested in the city of Ebolowa, Cameroon, and identified in the Cameroon National Herbarium, where the voucher specimen was kept under the reference numbers 42417/HNC. This plant was chosen on the basis of its traditional use in the treatment of bacterial infections, including respiratory tract infections. The plant was dried at room temperature (20-25 °C) and protected from light until a constant weight was obtained, then ground into powder in a mixer.

Preparation of the plant extracts

The powder obtained after grinding was macerated in ethanol for 48 hours and stirred several times a day to maximize the yield [8]. After 48 hours, the mixture was filtered using Whatman No. 1 paper, and the filtrate obtained was evaporated using a rotary evaporator at 65 °C. The extract was collected in a dry and sterile bottle and then placed in an oven at 40 °C until the solvent had completely evaporated. The extraction yield of the plant was calculated relative to the mass of the dry plant powder [8].

Chemical analysis of extracts

Qualitative Chemical Analysis of the Plant Extract

The main classes of compounds (alkaloids, flavonoids, anthraquinones, tannins, saponins, anthocyanins, triterpenes, phenols, polyphenols, and sterols) were detected in the *A. digitata *stem bark extract using standard methods [9].

Gas Chromatography and Mass Spectroscopy (GC-MS) Analysis and Identification of Bioactive Compounds

GC-MS analysis of biologically active compounds present in the *A. digitata *stem bark extract was performed at the Central Instrumental Laboratory (CIL) in NIFTEM College, Thanjavur, Tamil Nadu, India. GC-MS analysis of the extracts was performed using a GC-MS system (GC Model: 8890GC/5977B GC/MSD; Agilent Technologies, Santa Clara, CA) [10]. It is equipped with an Rtx-SMS column (5% diphenyl/95% dimethylpolysiloxane; 30 m × 0.25 mm ID × 0.25 µm df). The volume of sample injected was 2 µL. The helium was used as carrier gas, and its rate was set to 1 mL/minute. The split ratio was 10:1. The oven temperature program was 110 °C hold for 350 minutes, up to 200 °C at the rate of 10 °C/minute, no hold; up to 280 °C at the rate of 5 °C/minute for 12 min hold. The Injector temperature was 280 °C, and the total GC running time was 40.50 minutes. The Mass scan (m/z) of the spectrometer was set to 50-550 au. The oven temperature was set to 290 °C, and the detector temperature was set to 250 °C. The solvent delay was 0 to 3.5 minutes, and the total MS running time was 40.50 minutes. For GC-MS detection, an electron ionization system with an ionization power of 70 eV was used. The time from injection (start time) to the time at which the eruption occurred is referred to as the retention time (RT). The identity of the bio-constituents present in the A. digitata extract was determined by comparing their retention times and mass spectral fragmentation patterns with those in the NIST Library database (Version 2020), using MassHunter Workstation software (Agilent Technologies, Santa Clara, CA).

Antibacterial assays

Bacteria

The bacteria used in this work consisted of clinical isolates and reference strains of American Type Culture Collections (ATCC) of *Klebsiella pneumoniae*, *Klebsiella oxytoca*, *Pseudomonas aeruginosa, *and *Streptococcus pneumoniae*. These bacterial species were maintained in the research unit of Microbiology and Antimicrobial Substances in a mixture of glycerol and Mueller Hinton Broth (MHB) (1:1) at -4 °C, and activation was done with the streak technique on agar medium before any antibacterial test [8].

Preparation of Bacterial Inocula

The desired bacterial concentration for the test was prepared at 10^6^ colony-forming units (CFU)/mL from overnight cultures [11].

*In Vitro* *Antibacterial Assay*

The Minimum Inhibitory Concentrations (MIC) and Minimum Bactericidal Concentrations (MBC) of the plant extract were determined by the microdilution method [11]. Bacterial growth was monitored colorimetrically using iodonitrotetrazolium chloride (INT). The MIC was the lowest concentration of plant extract that prevented the change of INT color. The lowest concentrations that would induce an absence of colonies or the appearance of a colony only on the subculture dishes were considered to be the MBC. A plant extract with an MBC/MIC ratio ≤ 4 was described as bactericidal, while an extract with an MBC/MIC ratio strictly ˃4 was described as bacteriostatic [11]. Amoxicillin (Sigma-Aldrich, Sternheim, Germany) and Augmentin (amoxicillin/clavulanic acid (8/1), Sigma-Aldrich) were used as reference antibiotics. Three repetitions were carried out per test solution and per concentration.

Evaluation of the interaction of antibiotics with the extract

The interaction between the ethanol extract of the *A. digitata *stem bark and Augmentin was performed by using the Checkerboard microdilution method [12]. All the experiments were performed in triplicate.

In Vitro Antibacterial Mechanism Studies

The inhibition of the bacterial ATPases/H+ proton pump, outer membrane permeability, dehydrogenase activity, and leakage of nucleic acid assays were used to determine the mode of antibacterial action following the methods previously reported [8].

In Vivo Antibacterial Assay

Animals: For this study, 48 albino rats (males) of the *Wistar *strain aged 9 weeks were randomly distributed in cages into six groups of eight animals each, including a control group (G0) and five test groups (G1-G5). The mean initial weights of the animals of the different groups were comparable (*P* ≥ 0.05). Water and a standard laboratory diet were given *ad libitum* to the animals. Rats were acclimatized to experimental conditions (room temperature, cage grouping, 12 hours dark and 12 hours light, nutrition *ad libitum*) for one week at the animal house of the Department of Biochemistry of the University of Dschang. All the procedures and protocols involving animals and their care were conducted in conformity with the institutional guidelines and approved by the Cameroon National Ethical Committee (Reg. No. FWA-IRB00001954). Efforts were also made to minimize animal suffering and to reduce the number of animals used in the experiment.

Experimental design: The induction of *K. pneumoniae* infection was done through the nasal cavity of the animal [2]. Before infection, animals were fasted for 12 hours but had access to tap water *ad libitum*. All animals were anesthetized by inhalation of isoflurane and placed in a head-up, upright position. Then, 30 μL of suspension of *K. pneumoniae *(1.5 x 10^8^ CFU/mL) was inoculated into the animals through the nasal cavity. After inoculation, the rats were held with their heads upright for 20 seconds to ensure that the suspension indeed entered the lungs. Finally, the animals were housed in their respective cages. Four days after infection, *A. digitata* stem bark extract was administered orally once daily for seven consecutive days. The daily therapeutic dose was obtained from the MIC value [13]. Group G0 was taken as a neutral control and was not infected or treated. Group G1 was taken as a negative control and was infected and not treated. Group G2 was taken as a positive control and was infected and treated with the standard antibiotic (Augmentin, 1 mg/kg of body weight). Groups G3, G4, and G5 were infected and treated with the extract at dose 1 (3 mg/kg of body weight), dose 2 (5 mg/kg of body weight), and dose 3 (9 mg/kg of body weight), respectively.

Sacrifice of Animals and Collection of Blood and Organs

Animals were anesthetized by intraperitoneal injection of diazepam and ketamine (0.2/0.1 mL per 100 g), then placed in dorsal decubitus on a board, and the four limbs were immobilized to allow easy access to the abdomen. A dissection was performed on the abdomen, and blood was collected by cardiac puncture and then introduced into the tubes without anticoagulant for the detection of biological indices and organs such as The heart, lungs, liver, spleen, and kidneys were collected. The tubes were allowed to stand for one hour in ice and centrifuged at 3500 rpm for 15 minutes to obtain the serum.

Microbiological Evaluation

Approximately 0.5 g of lung tissue was cut and ground in a porcelain mortar with 4 mL of sterile physiological saline solution (0.9% NaCl), and the resulting homogenate was centrifuged at 3,000 rpm for 15 minutes. Then, the supernatant was decanted and used for culture on MacConkey agar to count the number of CFU of *K. pneumoniae* that were compared between the study groups. The presence of colonies on the fourth day post-infection testified to the establishment of the infection, while the cancellation of the bacterial load after lung culture of the animals on the seventhday post-treatment testified to the effectiveness of the treatment.

Evaluation of Biochemical Parameters

The sera prepared were subsequently used for serum assays of biological indexes, namely C-reactive protein (CRP), alanine aminotransferase (ALT), aspartate aminotransferase (AST), total protein (TP), and creatinine following the instructions of different commercial kits, and we randomly selected four rats in each group for pulmonary histopathological examination. AST and ALT were assayed using the Spinreact kit (Spinreact, Girona, Spain). Total serum protein was assayed by the Biuret method, using the SGM kit (SGM Italia, Rome, Italy). CRP was determined using a Monlab kit (Monlab, Barcelona, Spain). Serum calcium was assayed using the Arsenazo method kit (Biorex Diagnostics, Antrim, UK). Serum creatinine was measured by Jaffé's modified method using a Randox kit (Randox Laboratories, Mumbai, India).

Histopathological Analysis

This was carried out on lung, liver, and kidney tissue sections. After euthanasia, all animals were necropsied, and major organs, such as the lung, liver, and kidney, were surgically removed and fixed in 10% formalin in normal saline. Sections of 5 μm were obtained on a rotary microtome, and the material was stained with hematoxylin-eosin (HE) [14]. The stained slides of the sections from the 24 test animals were then analyzed using a microscope with a built-in digital camera (EVOS XL, Thermo Fisher Scientific, Waltham, MA) under 40× objective magnification to detect any abnormalities. The histology of the treated groups was compared with that of the neutral and negative control groups. Upon examination, the photomicrographs of the lungs, liver, and kidneys selected and depicted in the paper represent the general appearance observed in at least three of the four animals in the group.

Statistical analysis

Each parameter was assayed three times, and the resulting data were subjected to the analysis of variance (ANOVA) using the SPSS 23.0 software for Windows (IBM Corp., Armonk, NY). The Waller-Duncan test was used to compare the means of the different groups. A *P*-value < 0.05 was considered statistically significant.

## Results

Chemical analysis of the plant extract

The phytochemical analysis of the *A. digitata* stem bark extract showed the presence of flavonoids, phenols, sterols, triterpenes, tannins, alkaloids, anthocyanins, anthraquinones, and saponins.

GC-MS detected potential phytochemicals in the ethanol extract of *A. digitata* stem bark by their molecular formula and retention time. Twenty phyto-constituents with various pharmacological properties were detected by GC-MS (Table 1). The compounds identified in highest concentrations in the stem bark extract included 9,12-octadecadienoic acid (Z,Z) (18.62%), 9,12,15-octadecatrienoic acid (Z,Z,Z) (11.40%), *n*-hexadecanoic acid (9.35%), beta-amyrone (4.80%), 3-O-methyl-D-glucose (4.56%), squalene (4.50%), vitamin E (3.76%), capsaicin (2.81%), Z-8-methyl-9-tetradecenoic acid (2.71%), and 9,12-octadecadienoic acid (Z,Z), methyl ester (2.28%) (Table 1).

**Table 1:** Phytochemical constituents identified in the ethanol stem bark extract of Adansonia digitata by GC-MS analysis. RT, retention time; GC-MS, gas chromatography and mass spectroscopy

RT (minutes)	Compound names	Molecular formula	Peak area (%)
10.7742	Dodecanoic acid, 3-hydroxy-	C_12_H_24_O_3_	1.46
10.9229	Z-8-methyl-9-tetradecenoic acid	C_15_H_28_O_2_	2.71
12.1360	3-O-methyl-D-glucose	C_7_H_14_O_6_	4.56
13.0573	2-Hydroxy-5-methylisophthalaldehyde	C_9_H_8_O_3_	1.94
13.4406	2-Hexadecen-1-ol, 3,7,11,15-tetramethyl-, acetate	C_22_H_42_O_2_	1.19
14.4592	Hexadecanoic acid, methyl ester	C_17_H_34_O_2_	1.22
14.9284	n-Hexadecanoic acid	C_16_H_32_O_2_	9.35
15.3461	Trans-sinapyl alcohol	C_11_H_14_O_4_	0.27
16.6507	9,12-Octadecadienoic acid (Z,Z)-, methyl ester	C_19_H_34_O_2_	2.28
17.2515	9,12-Octadecadienoic acid (Z,Z)-	C_18_H_32_O_2_	18.62
17.3374	9,12,15-Octadecatrienoic acid, (Z,Z,Z)-	C_1_8H_30_O_2_	11.40
17.5605	Octadecanoic acid	C_18_H_36_O_2_	1.21
23.2082	Capsaicin	C_18_H_27_NO_3_	2.81
26.9103	Squalene	C_30_H_50_	4.50
31.3106	Vitamin E	C_29_H_50_O_2_	3.76
34.9612	Gamma-sitosterol	C_29_H_50_O	1.88
36.2029	Beta-amyrone	C_30_H_48_O	4.80
36.8152	Alpha-amyrin	C_30_H_50_O	1.53
38.4803	Gamma-sitostenone	C_29_H_48_O	1.37

*In vitro* antibacterial activity of the *A. digitata* extract

The* in vitro* antibacterial activity was evaluated through the determination of MICs of the plant extract against multi-drug-resistant pathogenic bacteria. The results showed that the *A. digitata* extract displayed distinct antibacterial activities against the tested bacteria, with MICs ranging from 64 to 2,048 µg/mL (Table 2). The lowest MIC values (indicating the highest antibacterial activity) were observed against *K. pneumoniae* 22 and *K. pneumoniae* 106. In contrast, the highest MIC values (indicating the lowest antibacterial activity) were recorded for *K. pneumoniae* 26, 31, 44, 46, and *K. oxytoca*. Most of the MBC values were obtained with this plant extract, suggesting its bactericidal effect. With regard to Table 2, the studied bacteria were shown to be more resistant to amoxicillin with MICs greater than 256 μg/mL compared to Augmentin (MIC = 8-256 μg/mL).

**Table 2:** Antibacterial activity (MIC and MBC in µg/mL) of the ethanol stem bark extract of Adansonia digitata according to the tested bacteria. MIC, minimum inhibitory concentration; MBC, minimum bactericidal concentration

Bacteria	*A. digitata* (stem bark)		Augmentin		Amoxicillin	
	MIC	MBC	MIC	MBC	MIC	MBC
*Pseudomonas aeruginosa* 21	128	256	128	128	128	> 256
*Klebsiella pneumoniae *22	64	256	16	64	64	128
*K. pneumoniae* 26	1024	2048	64	64	64	128
*K. pneumoniae *31	1024	2048	128	256	> 256	> 256
*K. pneumoniae* 44	1024	2048	16	32	64	128
*K. pneumoniae* 46	1024	> 2048	16	64	128	128
*K. pneumoniae *56	512	2048	64	128	256	> 256
*K. pneumoniae* 106	64	128	8	32	64	256
*K. oxytoca* 26	512	1024	16	32	256	>256
*K. oxytoca* 43	1024	>2048	256	>256	256	>256
*K. oxytoca* 107	512	512	256	>256	>256	>256
*Streptococcus pneumoniae* ATCC 49619	256	1024	32	128	64	128
*K. pneumoniae* ATCC 10031	128	128	64	128	128	256

Effect of the combination of the extract and Augmentin

*A. digitata* extract and Augmentin, when used in combination, exerted an indifferent effect against *K. pneumoniae* 26 and *Streptococcus pneumoniae* ATCC 49619; an additive effect against *Pseudomonas aeruginosa* 21, *K. pneumoniae* 22, 31, 44, 46, and *K. oxytoca* 43; and a synergistic effect against *K. pneumoniae* 56, 106, 26, *K. oxytoca* 107, and *K. pneumoniae* ATCC 10031 (Table 3).

**Table 3:** Fractional inhibitory concentrations (FICs) calculated for the combination of Augmentin and Adansonia digitata extract against the studied bacterial strains. ∑FIC, sum of fractional inhibitory concentrations

Bacteria	∑FIC	Interpretation
*Pseudomonas aeruginosa* 21	0.50	Additive
*Klebsiella pneumoniae* 22	0.62	Additive
*Klebsiella pneumoniae* 26	1.00	Indifference
*Klebsiella pneumoniae* 31	0.75	Additive
*Klebsiella pneumoniae* 44	0.75	Additive
*Klebsiella pneumoniae* 46	0.50	Additive
*Klebsiella pneumoniae* 56	0.31	Synergy
*Klebsiella pneumoniae *106	0.18	Synergy
*Klebsiella oxytoca* 26	0.37	Synergy
*K. oxytoca* 43	0.75	Additive
*K. oxytoca* 107	0.37	Synergy
*Streptococcus pneumonia*e ATCC 49619	1.00	Indifference
*K. pneumoniae* ATCC 10031	0.25	Synergy

Mechanisms of the antibacterial activity

**Figure 1:**
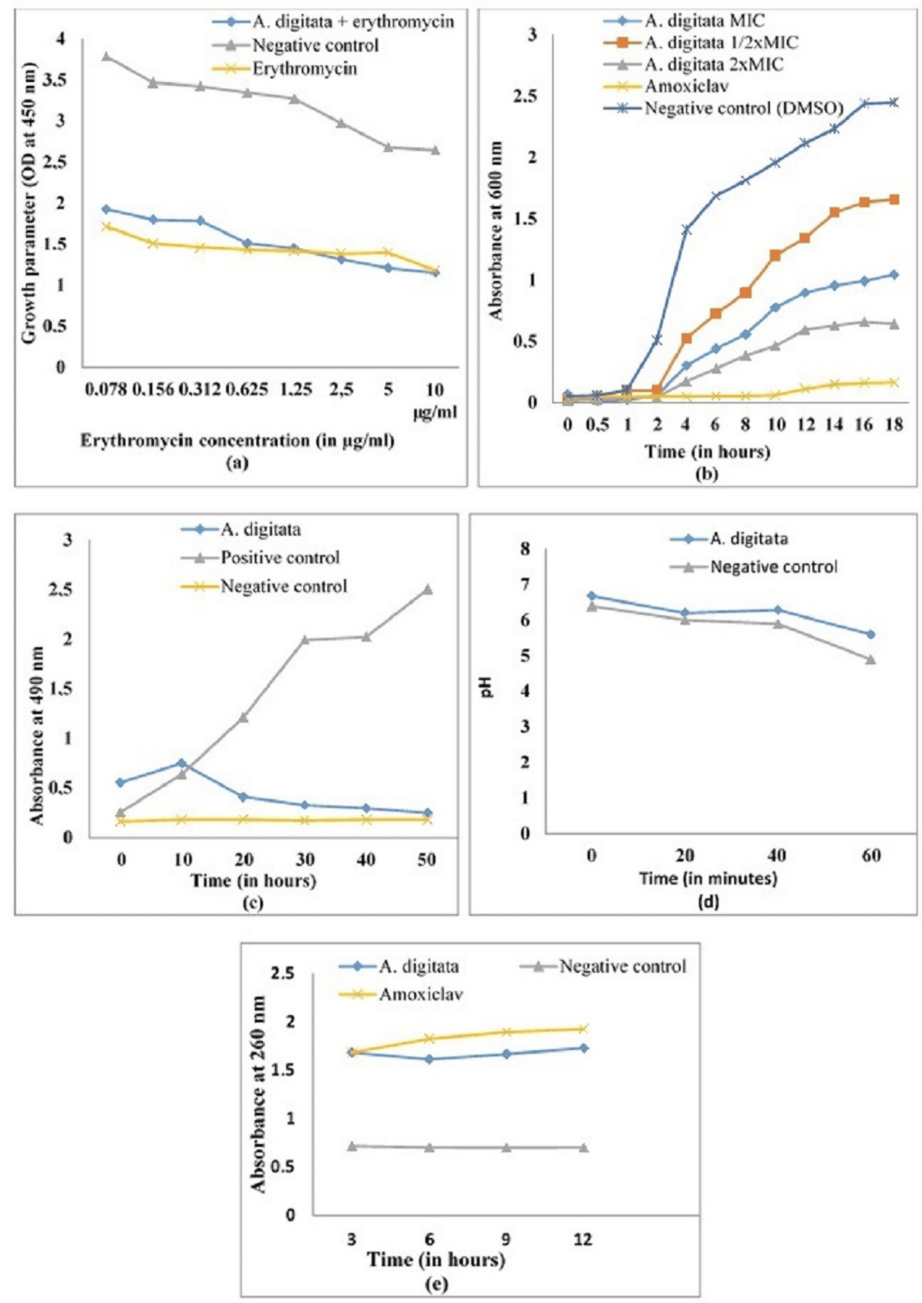
Effect of the A. digitata extract on (a) outer membrane permeability, (b) growth kinetics, (c) dehydrogenase activity, (d) ATPase/H⁺ proton pump function, and (e) nucleic acid leakage in K. pneumoniae 106. The results are expressed as mean ± standard deviation of three repetitions. DMSO, dimethyl sulfoxide; MIC, minimum inhibitory concentration

The co-incubation of erythromycin with bacterial suspension had a measurable inhibitory effect on the *K. pneumoniae* growth (Figure 1a). The combination of the *A. digitata *extract with erythromycin caused *K. pneumoniae* 106 to be more susceptible to the erythromycin at the tested concentrations of 2.5, 5, and 10 µg/mL (Figure 1a). The potentiating effect of the *A. digitata* extract on the membrane permeability to erythromycin was not observed against *K. pneumoniae *106 at the concentrations of erythromycin less than 2.5 µg/mL, where the effect of the erythromycin + *A. digitata* extract was less than that of the erythromycin alone (Figure 1a).

The growth kinetic curves of *K. pneumoniae* showed that the curve of *K. pneumoniae* in the presence of dimethyl sulfoxide (DMSO) presents four phases with a final optical density of 2.4 (Figure 1b). At different concentrations (MIC, MIC/2, and 2MIC) of the *A. digitata *extract, a considerable decrease in cell growth is observed with a lower final optical density for the concentrations of MIC and 2MIC compared to MIC/2. *A. digitata* extract displayed all the phases of the bacterial growth: a lag phase (0-2 hours), an acceleration phase (2-8 hours), an exponential phase (8-16 hours), and a stationary phase (16-18 hours). No considerable growth is observed in the presence of Augmentin; hence, an almost constant lag phase until the end of the experiment (Figure 1b).

The activity of respiratory chain dehydrogenases in untreated healthy *K. pneumoniae* cells (positive control) increased with incubation time (Figure 1c). However, this activity underwent virtually no change in tubes containing cells heated at 100 °C for 20 minutes (negative control). The enzymatic activity of *K. pneumoniae* cells treated with the 64 μg/mL extract remained significantly lower than that of the positive control during the 40-minute incubation.

The pH variation curves as a function of time of the reaction medium containing *K. pneumoniae* cultures treated with the extract of *A. digitata *showed slight decreases in the pH values ​​of the medium during the first 20 minutes with the extract compared to the control (Figure 1d). For 40 minutes, the effect of the extract remained significantly (*P* < 0.05) greater compared to that of the negative control.

A significant leakage of nucleotides is observed from the bacteria treated with *A. digitata* extract as a function of incubation time compared to the negative control (Figure 1e). The effect observed with the *A. digitata* extract is equal to that of Augmentin at the third hour of incubation. From the sixth hour to the 12th hour of incubation, Augmentin caused the leakage of the greatest amount of nucleotide.

*In vivo* anti-*Klebsiella pneumoniae *activity of the *A. digitata* extract

Effect of Different Doses of the A. digitata Extract on the Bacterial Load (CFU/mg) in the Lungs of Infected Rats

In all the infected groups, no significant difference was observed between the bacterial loads per mg of lungs after four days post-infection, whereas no bacterial colony was found after seven days post-treatment in the neutral control (Table 4). However, in infected and untreated animals (negative control), 82.67 × 10³ CFU/mg of bacteria were detected in the lungs. The bacterial load in the lungs of the negative control group was significantly higher (*P* < 0.05) compared to that observed in animals treated with the *A. digitata* stem bark extract at dose 1 (3 mg/kg), suggesting *in vivo* anti-*K. pneumoniae* activity. Interestingly, dose 2 (5 mg/kg) and dose 3 (9 mg/kg) of the *A. digitata* stem bark extract, as well as Augmentin (1 mg/kg), eradicated all *K. pneumoniae* after seven days post-treatment.

**Table 4:** Number of colony-forming units of Klebsiella pneumoniae per mg of lung (CFU/mg) on the fourth day post-infection and the seventh day post-treatment with Adansonia digitata extract. Data are expressed as mean ± standard deviation, *n* = 4; values in the same column followed by different superscript letters (a, b, c) are significantly different according to Waller-Duncan’s multiple comparison test (*P* < 0.05).

Groups	Number of bacterial colonies x 10³ CFU/mg of lung	
	Fourth day post-infection	Seventh day post-treatment
Neutral control	0.00 ± 0.00ª	0.00 ± 0.00ª
Negative control	266.67 ± 76.37ᵇ	82.67 ± 14.57ᵇ
Positive control (Augmentin)	283.33 ± 28.86ᵇ	0.00 ± 0.00ª
Dose 1 (3 mg/kg)	280 ± 50.00ᵇ	8.33 ± 2.08^c^
Dose 2 (5 mg/kg)	266.67 ± 28.86ᵇ	0.00 ± 0.00ª
Dose 3 (9 mg/kg)	266.67 ± 28.86^b^	0.00 ± 0.00ª

Effect of Treatment on the Biochemical Profile of Animals

On the fourth day post-infection, *K. pneumoniae* infection resulted in a significant increase (*P* < 0.05) in biochemical parameters: ALT, AST, total protein, creatinine, and CRP compared to the neutral control (Table 5). Interestingly, on the seventh day post-treatment, *A. digitata* extract (at the dose of 3 mg/kg of body weight) resulted in a significant (*P* < 0.05) improvement in biochemical parameters compared to the negative group (infected and untreated group). In addition, on the seventh day post-treatment, the plant extract (at doses of 5 and 9 mg/kg body weight) and Augmentin led to normalization of biochemical parameters compared to the negative control group (infected and untreated).

**Table 5:** Evolution of the selected biochemical markers in animals on the fourth day post-infection and the seventh day post-treatment. Data are expressed as mean ± standard deviation, *n* = 4; values for a given biochemical parameter followed by a different letter as superscript (a, b, c, d, e, f) are significantly different according to Waller–Duncan’s multiple comparison test (*P* < 0.05). 4DPI, fourth day post-infection; 7DPT, seventh day post-treatment

Groups	Alanine aminotransferase (ALT)		Aspartate aminotransferase (AST)		Total protein		Creatinine		Calcium		C-reactive protein	
	4DPI	7DPT	4DPI	7DPT	4DPI	7DPT	4DPI	7DPT	4DPI	7DPT	4DPI	7DPT
Neutral control	15.86 ± 4.99ª	11.94 ± 0.74ª	22.75 ± 11.60ª	17.83 ± 4.34ª	1.96 ± 0.64^a^	2.53 ± 0.02^a^	0.83 ± 0.45ª	0.74 ± 0.73ª	7.44 ± 0.42ª	6.80 ± 1.15ª	7.42 ± 2.33 ª	7.26 ± 1.14ª
Negative control	78.86 ± 10.54ᵇ	89.63 ± 3.24^d^	100.41 ± 4.35^e^	79.92 ± 7.89 ͨ	6.84 ± 0.57d	6.74 ± 0.42^c^	1.36 ± 0.80^ab^	2.90 ± 1.34ᵇ	7.15 ± 0.23ª	7.37 ± 0.66ª	19.80 ± 4.98ᵇ	22.00 ± 3.81ᵇ
Positive control (Augmentin)	71.56 ± 10.35ᵇ	11.79 ± 1.19ª	94.17 ± 11.03^ef^	16.63 ± 2.62ª	7.07 ± 0.47ᵈ	2.57 ± 0.47ª	1.03 ± 1.23^ab^	1.16 ± 0.18ª	6.41 ± 0.83ª	6.93 ± 1.01ª	23.92 ± 4.91ᵇ ͨ	7.70 ± 1.90ª
Dose 1 (3 mg/kg)	72.56 ± 9.82ᵇ	14.81 ± 2.80ª	82.54 ± 14.63^ef^	42.58 ± 23.88ᵇ	6.77 ± 0.16^d^	3.54 ± 0.34ᵇ	1.26 ± 0.75^ab^	1.05 ± 0.24ª	6.80 ± 0.59ª	7.47 ± 0.83^ab^	24.20 ± 6.75ᵇ ͨ	8.80 ± 3.81ª
Dose 2 (5 mg/kg)	79.75 ± 6.80ᵇ	14.49 ± 4.13ª	86.13 ± 7.48^f^	21.29 ± 7.44ª	6.91 ± 0.49^d^	3.27 ± 0.49ᵇ	1.63 ± 0.71^ab^	1.47 ± 1.19ªᵇ	7.51 ± 0.91ª	6.77 ± 0.49ª	28.05 ± 5.85 ͨ	8.80 ± 3.81ª
Dose 3 (9 mg/kg)	115.99 ± 11.89 ͨ	12.22 ± 0.58ª	91.38 ± 4.69^ef^	10.50 ± 1.75^d^	6.86 ± 0.34^d^	2.25 ± 0.34ª	2.17 ± 1.46^ab^	0.90 ± 0.48ª	6.71 ± 0.94ª	8.50 ± 0.50^b^	21.68 ± 4.61ᵇ	11.00 ± 3.81ª

Effect of Infection and Treatment on the Histology of the Lung of Animals on the Fourth Day Post-Infection and Seventh Day Post-Treatment

**Figure 2:**
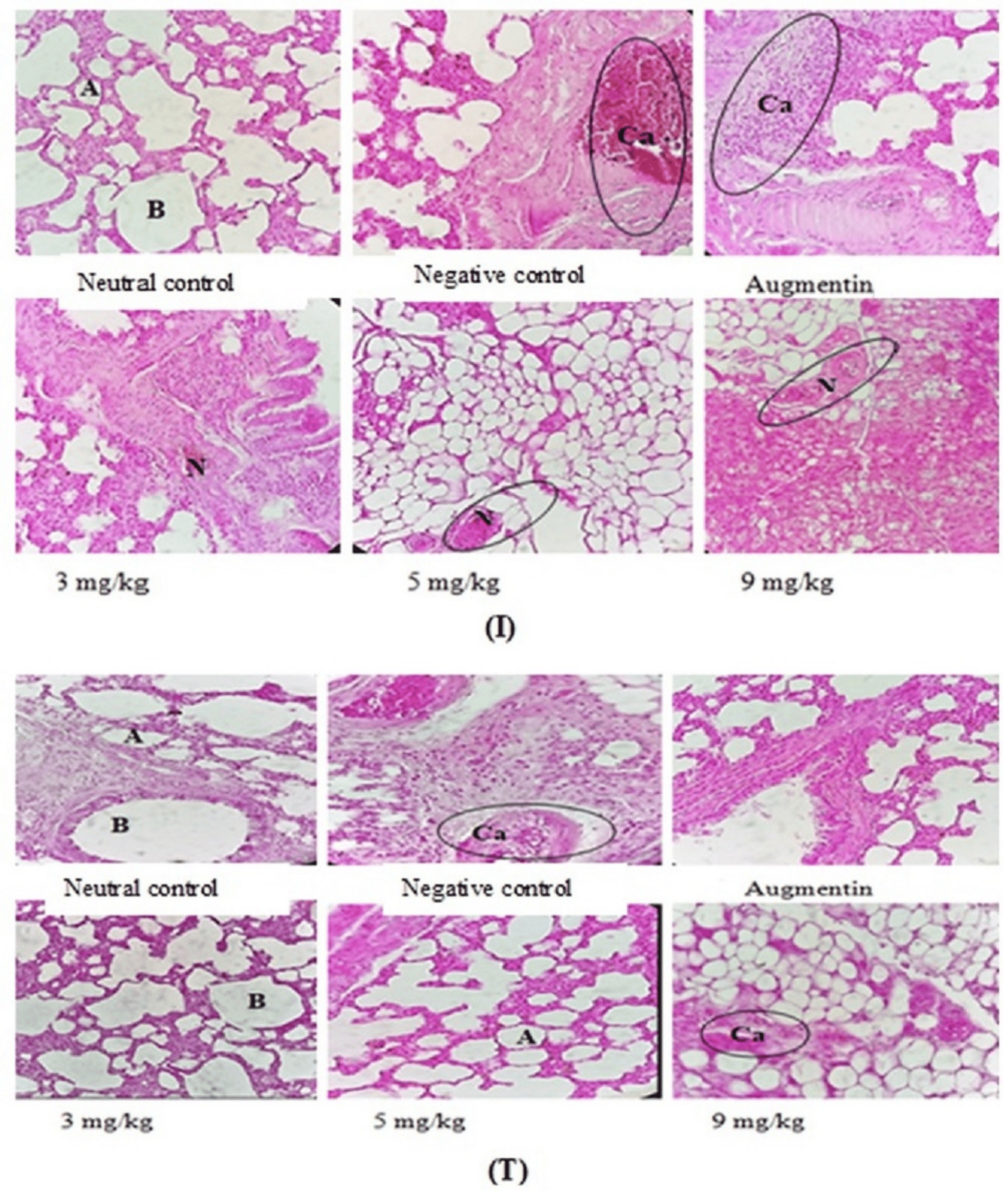
Histological features of the lungs in male rats (H&E, ×400) on the fourth day post-infection (I) and the 11th day post-treatment (T). A, alveolus; B, bronchi; N, necrosis; Ca, Carcinoma; H&E, hematoxylin and eosin

Figure 2 shows the lung sections of control animals and those treated with* A. digitata* extract on the fourth day post-infection and seventh day post-treatment. It appears that the infection caused changes in the lungs of infected animals on the fourth day post-infection, marked by the presence of necrotizing inflammation and dilation of tissue sinusoids. Interestingly, daily administration of *A. digitata* extract and Augmentin for seven days resulted in a correction of these abnormalities observed in the lungs of infected animals, suggesting that *A. digitata* extract and Augmentin eradicated *K. pneumoniae* infection in the lungs of infected animals. These abnormalities remained in negative control (i.e., infected and untreated animals) on the seventh day post-treatment (Figure 2).

## Discussion

In this study, the qualitative chemical analysis of the *A. digitata* extract was carried out with the aim of determining the various classes of secondary metabolites, which could explain their antibacterial properties. Indeed, the antibacterial activity of medicinal plants is correlated with the presence and level of one or more classes of bioactive secondary metabolites [11]. Similar to our results, early studies demonstrated the presence of phenols, flavonoids, and tannins in the stem bark extract of* A. digitata* [5] and the antibacterial properties of *α*-amyrin, beta-amyrone, octadecanoic acid, 9,12,15-octadecatrienoic acid, *n*-hexadecanoic acid, 2-hydroxy-5-methylisophthalaldehyde, and dodecanoic acid found in the *A. digitata* extract [15-17]. According to the cutoff values of MICs established by Tamokou et al. [18], the *A. digitata* stem bark extract was highly active (MIC < 100 μg/mL) against *K. pneumoniae* 22 and *K. pneumoniae* 106; significantly active (100 < MIC ≤ 512 μg/mL) against *P. aeruginosa* 21, *K. pneumoniae* 56, *K. oxytoca* 26, *K. oxytoca* 107, *S. pneumoniae* ATCC 49619, *K. pneumoniae* ATCC 10031; moderately active (512 < MIC ≤ 2048 μg/mL) against *K. pneumoniae* 26, 31, 44, 46, and *K. oxytoca* 43. The differences in sensitivity observed between different bacterial species may be due to genetic variability between the strains [11]. The findings of this study indicate that most of the MBC/MIC ratios are lower than or equal to 4, indicating that the *A. digitata* stem bark extract generally has a bactericidal effect [11].

The effect of the association of the *A. digitata* stem bark extract with Augmentin was additive against *P. aeruginosa* 21, *K. pneumoniae *22, 31, 44, 46, and *K. oxytoca* 43; synergistic against *K. pneumoniae *56, 106, 26, *K. oxytoca *107, and *K. pneumoniae* ATCC10031. These findings indicate increased bacterial susceptibility to the test antibiotics in the presence of the *A. digitata* extract, suggesting that the combination of *A. digitata* extract with antibacterial drugs could be an alternative to treat invasive bacterial infections involving drug-resistant *Klebsiella* spp.

In this study, *A. digitata *extract resulted in a decrease in bacterial growth at concentrations below the MIC of erythromycin, suggesting the ability of the plant extract to facilitate the passage of erythromycin through the bacterial outer membrane. This membrane destabilization could explain the anti-*Klebsiella *activity of *A. digitata*. A significant shortening of the exponential growth phase of *K. pneumoniae *was recorded in the presence of *A. digitata *extract. However, a longer latency phase is observed in the presence of the extract compared to the control containing DMSO, suggesting partial deactivation of the cells and a reduction in the initial viable load; the bacteria need more time, along with newly synthesized proteins and enzymes, to be able to divide. This could justify the inhibition of the growth, particularly in the latency phase, by the *A. digitata* extract. The activity of the respiratory dehydrogenase in the *K. pneumoniae* treated with the extract was lower than that of the positive control, indicating that the bacterial electron transport chain was affected or even inhibited by the plant extract. The latter could inhibit bacterial growth by uncoupling the electron transport chain of bacteria. This uncoupling can either inhibit respiratory chain dehydrogenases by disrupting oxidative phosphorylation or destroy them, with the consequence of inhibiting cell respiration [19]. According to the results obtained, the decrease in the pH of the medium containing the bacterial cells treated with the *A. digitata* extract and the control demonstrates the inhibition of the ATPase/H+ proton pumps of *K. pneumoniae*. This result suggests that some secondary metabolites contained in the plant extract have ATPase/H+ proton pumps as their target of action. The addition of the *A. digitata* extract resulted in a significant leakage of *K. pneumoni*a nucleotides. This leakage suggests the loss of bacterial membrane integrity and, consequently, the death of the bacteria. The leakage of intracellular materials from cells is an indicator of major damage to the bacterial cell membrane [19].

Based on information provided by the *in vitro* antibacterial test results, an *in vivo* study was undertaken with a view to demonstrating the therapeutic efficacy of the *A. digitata* stem bark extract against *K. pneumonia* infection in a rat model. In case of pneumonia, the alveoli are filled with pus and fluid, which makes breathing painful. Several microorganisms cause this pneumonia, such as *K. pneumoniae*, which is the main bacterial species responsible for intra-hospital epidemics. It is an opportunistic pathogen, multi-resistant to antibiotics, responsible for infections linked to care, often following colonization of the digestive tract. Studies have shown that the pathogenicity and virulence of *Klebsiella *strains are host-specific [20]. Thus, *K. pneumoniae* induces systemic infection in humans and some animals, namely, rabbits and rats [21]. Several important factors of *Klebsiella *infection, such as intestinal transport, electrolyte transport, and colonization of the ileum, are similar in rats and humans [21], justifying the choice of *Wistar *albino rats for this study. Mammals, and particularly rats, are the most widely used models to study *K. pneumoniae* infection. Anatomical similarities and comparable immune responses, combined with ease of handling, make these animals the reference for *K. pneumoniae* infection. The increase in bacterial load after lung culture on the fourth day post-infection, associated with many other signs such as behavioral disorders, sneezing, anorexia, and especially the death of several animals, could reflect the installation of *K. pneumoniae* infection. The administration of the *A. digitata* extract led to a decrease in the bacterial load in the treated groups, with a complete cure observed in the animals treated at doses of 5 and 9 mg/kg on the seventhday post-treatment. The therapeutic effect of the *A. digitata* extract was manifested by the disappearance of the clinical signs (weight loss, death, lung inflammation) previously observed in the animals. These results clearly show that *A. digitata* extract possesses a therapeutic effect against pneumonia caused by *K. pneumoniae*.

The liver is the driving organ of metabolism. It recovers and transforms many toxins to render them harmless before elimination. A dysfunction of this organ would cause severe damage to the body. The measurement of the activity of transaminases such as AST and ALT made it possible to evaluate liver function. Indeed, the increase in serum transaminase activity and total serum protein content observed on the fourth day post-infection in this study is due to infection by *K. pneumoniae*. These increases could be due to liver damage resulting from the adverse effects of endotoxins (lipopolysaccharides) released by *Klebsiella, *which infected the Küpffer cells [21]. In acute sepsis, the liver is extensively affected, and enzymes and cytoplasmic proteins of hepatocytes can *leak* from liver cells into the blood due to increased membrane permeability [22]. However, the decrease in serum transaminase levels and total proteins observed in animals on the seventh day post-treatment compared to the negative control could reflect the corrective effect of the *A. digitata* extract on the lungs of rats. These results were further confirmed by the histopathological analysis of the lungs of the animals on the seventh day post-treatment, which revealed no abnormalities in the animals receiving the different doses of extracts. These results are in agreement with those of Kodjio et al. [23] who had shown that some plant extracts could remedy the liver damage caused by the infection. The extract at these doses would not only treat the infection caused by *K. pneumoniae* but also reportedly reverse the damage caused by the infection.

To attack them, *K. pneumoniae* adheres to the epithelial cells of the upper respiratory tract, gastrointestinal tract, and endothelial cells before colonizing the mucous membranes. The underlying conditions are often renal failure with complications such as pulmonary infection evolving into necrosis and disintegration affecting the entire lobe [24]. The kidneys represent the main system of excretion of medicinal substances as well as their various metabolites. The levels of creatinine and total proteins allow for the evaluation of kidney function. The increase in creatinine, even if not statistically significant, and the elevated level of total protein observed in animals on the fourth day post-infection compared to the neutral controls may reflect renal dysfunction caused by the infection, potentially disrupting glomerular filtration. These results show that the infection would have caused renal dysfunction. This corroborates the findings of Méric et al. [25], who showed that Klebsiella infection is associated with renal damage. This infection may have either caused glomerulonephritis, as according to Aliyu et al. [26], each marker of nephrotoxicity (creatinine) increases significantly in the serum only if 75% of the nephrons have been destroyed; or caused an acceleration in the rate of degradation of creatinine synthesized by the liver and kidneys and then stored in the muscles. The curative effect was evidenced by the normalization of the creatinine and total protein levels on the seventh day post-treatment in the treated animals compared to the neutral and negative controls, which could be attributed to the antibacterial activity of the *A. digitata* extract. *A. digitata* extract is reported not only to treat *K. pneumoniae* pneumonia but also to help restore kidney architecture damaged by the infection.

In current clinical practice, CRP is a biomarker used mainly for diagnostic guidance and monitoring of diseases associated with a biological inflammatory syndrome [27]. It is known as a prognostic marker in many selected acute or chronic situations, such as pulmonary infection with *K. pneumoniae*. In the present study, the infection led to a significant increase in CRP on the fourth day post-infection, suggesting the inflammation in the lungs as revealed by the histopathological analysis. Interestingly, treatment with different doses of *A. digitata* extract normalized this parameter on the seventh day post-treatment compared to the neutral and negative controls. The results of the present study justify the traditional use of *A. digitata* in the treatment of respiratory tract infections, particularly those caused by *K. pneumoniae*. Furthermore, the results indicated that a *K. pneumoniae *infection significantly alters the biochemical parameters. To our knowledge, this is the first study to demonstrate the therapeutic properties of *A. digitata* stem bark extract and its synergistic effect with Augmentin.

Limitations

The development of a phytomedicine from *A. digitata* stem bark extract to treat pneumonia caused by *K. pneumoniae* requires clinical studies, investigation of additional antibacterial action sites, and the isolation and quantification of bioactive compounds.

## Conclusions

The results of this study demonstrate the antibacterial activity of *A. digitata* stem bark extract that could be attributed to the presence of flavonoids, phenols, sterols, triterpenes, tannins, alkaloids, anthocyanins, anthraquinones, and saponins. The plant extract induces changes in the bacterial growth kinetics, permeability of bacterial membranes, leakage of nucleic acids, and the inhibition of the bacterial ATPases/H+ proton pumps. Also, the findings of the present investigation show the synergistic and additive effects of *A. digitata* with Augmentin as well as its therapeutic efficiency on rats infected with *K. pneumoniae*. Hence, *A. digitata* can be used for the development of phytomedicines to treat respiratory tract infections, particularly those caused by the tested bacteria. So, further investigations, including clinical trials, are needed.
